# Evaluation of the delivery of an anti-*Listeria* endolysin via CRISPR-Cas9 engineered probiotic *Saccharomyces boulardii*

**DOI:** 10.1007/s00253-026-13749-6

**Published:** 2026-02-26

**Authors:** David Sáez Moreno, João Paulo Carvalho, Ellen Murray, Natalia Soledad Ríos Colombo, Alexandre Lamas, Alejandra Cardelle Cobas, Colin Hill, Joana Azeredo, Lucília Domingues

**Affiliations:** 1 CEB - Centre of Biological Engineering, University of Minho, Braga, Portugal; 2 LABBELS - Associate Laboratory, Braga, Guimarães, Portugal; 3 APC Microbiome Ireland, University College Cork, Cork, Ireland; 4 University of Santiago De Compostela, Lugo, Spain

**Keywords:** *Listeria monocytogenes*, Endolysin, CRISPR-Cas9, Biocontrol, Engineered probiotics

## Abstract

**Abstract:**

Listeriosis is a foodborne infection caused by *Listeria monocytogene*s that causes febrile gastroenteritis and central nervous system infections and that can often lead to fatality. Upon consumption of contaminated food, *Listeria* is able to survive a number of gastrointestinal stressors, including competition with the host microbiota. The emergence of antibiotic-resistant clones of *L. monocytogene*s, together with the side effects of antibiotic treatment, highlights the need for alternatives or additives for its treatment and prevention. *Saccharomyces boulardii* is a probiotic yeast that is often used alongside antibiotics to minimize side effects since it is not affected by them as a result of its eukaryotic nature. Furthermore, it can be engineered to produce a wide range of molecules. We previously engineered *Saccharomyces cerevisiae* through CRISPR-Cas9 integration to produce Ply511, a bacteriophage endolysin active against *L. monocytogene*s, showing the potential of engineered yeast to produce endolysins for biocontrol. In this study, we extended this approach to the probiotic yeast *S. boulardii* and directly compared the two yeasts as secretion hosts for Ply511. Using a simulated human gastrointestinal environment, we evaluated their ability to retain endolysin activity and reduce *L. monocytogenes* levels. We then tested the cell extracts from both yeasts in a bacterial consortium termed SImplified HUman intestinal MIcrobiota (SIHUMI), confirming a specificity for *Listeria*. Finally, we evaluated their activity in a simulated intestinal fermentation using fecal samples from human donors. Overall, this study demonstrates the potential of delivering endolysins to the gut via engineered probiotic *S. boulardii.*

**Key points:**

- *CRISPR-Cas9-engineered S. boulardii and S. cerevisiae were compared, both allowing the expression and activity of endolysin Ply511 against L. monocytogenes.*
- *Endolysin Ply511 retained its activity against L. monocytogenes in simulated gastrointestinal digestion and was specific against Listeria in a bacterial consortium termed SImplified HUman intestinal MIcrobiota (SIHUMI).*
- *Using fecal samples from human donors, the anti-Listeria effect was reduced potentially due to the lower metabolic activity of S. boulardii and the higher competition with the intestinal microbiome.*

**Graphical Abstract:**

**Figure:**
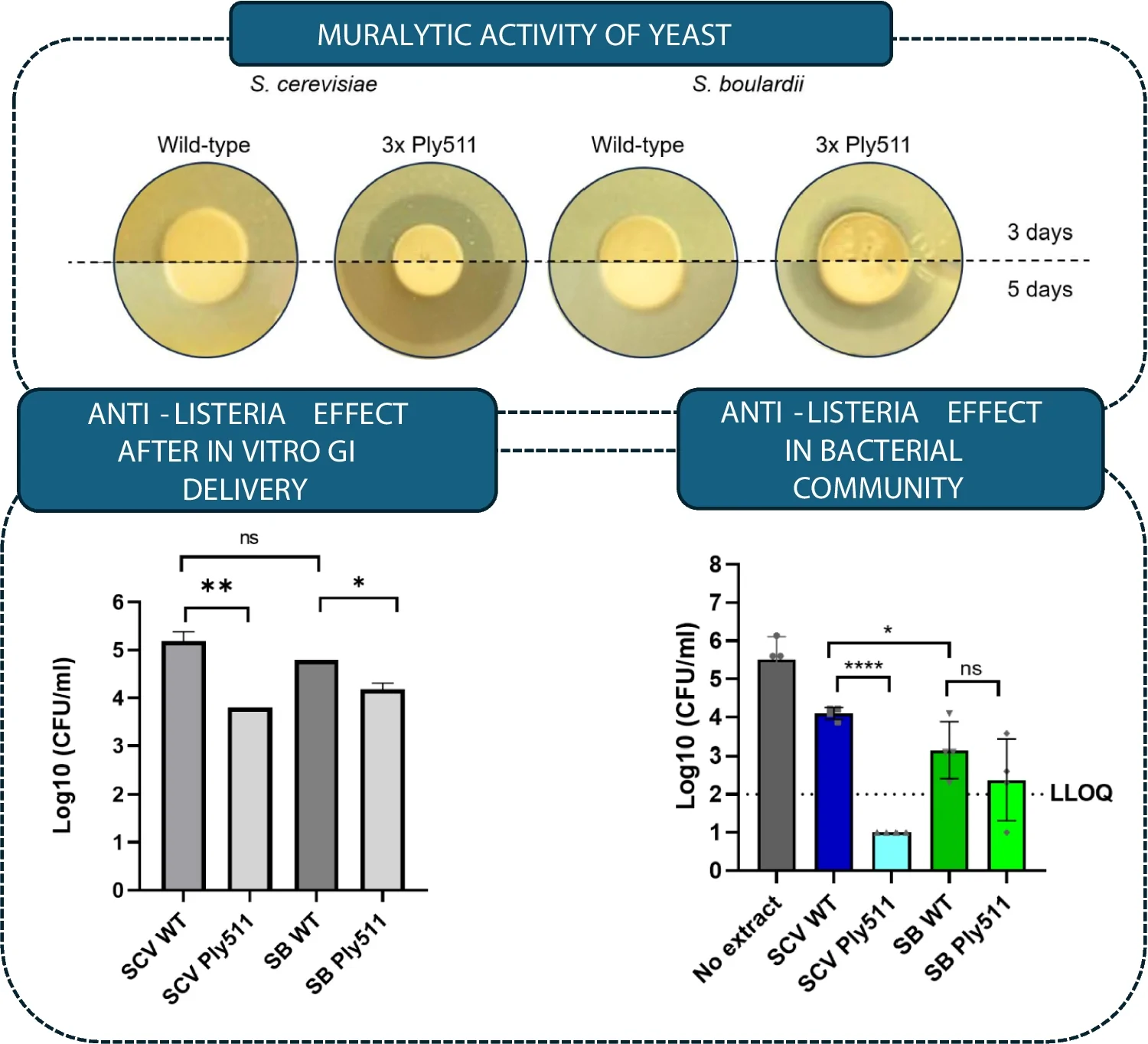

**Supplementary Information:**

The online version contains supplementary material available at 10.1007/s00253-026-13749-6.

## Introduction

*Listeria monocytogenes* is a foodborne pathogen that causes listeriosis, a disease producing a range of symptoms from febrile gastroenteritis to meningitis, often resulting in fatalities (Amato et al. 2017; Brouwer and van de Beek (Brouwer, and Beek, 2023)). Upon consumption of contaminated food*,* the bacterium can survive gastrointestinal (GI) stressors such as mechanical digestion, saliva, the low stomach pH, enzymes, and bile acids (Gahan and Hill 2014; Oliveira et al. 2025). Once in the intestine, it competes with the commensal microbiota. *L. monocytogenes* may survive these interactions and translocate across the GI barrier to initiate its infection cycle, reaching the bloodstream (Luque-Sastre et al. 2018). Moreover, it has been found in human feces which indicates that, apart from its transient colonization for the initiation of infection, it can also reside for long periods of time in the gut (Hafner et al. 2021). The protective effect of a varied microbiome has been suggested due to the correlation of *L. monocytogenes* load in human fecal samples with a low α-diversity (Hafner et al. 2021). The variety of microbes in the gut decreases with antibiotic (Huang et al. 2022), which combined with the worldwide appearance of antibiotic-resistant clones of *L. monocytogenes* (Hanes and Huang 2022; Kayode and Okoh 2022), highlights the need for the development of alternatives to antibiotics for the treatment and prevention of *L. monocytogenes* infections.

Probiotics can confer health benefits upon ingestion, including enhancing protection against pathogens. An example of this protection is the production of nisin, a bacteriocin produced by *Lactococcus lactis* affecting *L. monocytogenes* (Field et al. 2023). The interest in modifying human host-microbiota systems for therapeutic purposes has led to the development of engineered live biotherapeutic products, or engineered probiotics, to enhance probiotic properties (Rutter et al. 2022). *Saccharomyces boulardii* is a probiotic that is often used alongside antibiotics to minimize posterior gut dysbiosis (Lau and Chamberlain 2016) since, due to its eukaryotic nature, it is not affected by antibiotics and it can be engineered to produce a wide range of molecules (Carvalho et al. 2025).

Endolysins, phage-derived antibacterials, also referred to as enzybiotics (Murray et al. 2021), have been proposed as an alternative or adjunct to antibiotics. Their main advantage over antibiotics to treat bacterial infections is the fact that they would be expected to have minimal impact on the host microbiome because of their high specificity (Pottie et al. 2024). Endolysin Ply511 is active against all serovars of *Listeria* spp. (Schmelcher et al. 2010; Eugster and Loessner 2012) and could be suitable for the prevention or treatment of listeriosis. To date, several studies have shown the production of different endolysins by different hosts, specifically *Saccharomyces cerevisiae*, and by bacterial probiotic strains (reviewed elsewhere: (Pottie et al. 2024)). However, we are not aware of any previous reports of engineering *Saccharomyces boulardii* to produce endolysins, nor of directly assessing the effect of these endolysins on the gut microbiome.

We previously engineered *S. cerevisiae* to express Ply511, an endolysin effective against *L. monocytogenes* (Moreno et al. 2025). In this study, we engineered *S. boulardii* to produce Ply511 and we evaluate its performance under simulation of gastrointestinal (GI) digestion. We also evaluated its effect in a SImplified HUman intestinal MIcrobiota (SIHUMI) model, a bacterial consortium composed of culturable and fully sequenced human-derived enteric strains that can be individually tracked using qPCR (Eun et al. 2014; Buttimer et al. 2022; Ríos Colombo et al. 2023), and finally we assessed its effect in a model of the fecal microbiome, upon simulated intestinal fermentation using samples from human donors.

## Materials and methods

### *Escherichia coli *transformation and maintenance

*E. coli* DH5/NZY5α (NZYtech, Lisbon, Portugal) was used as the host strain for plasmid assembly and propagation*.* Transformation was carried out according to manufacturer instructions (NZYtech, Lisbon, Portugal) and as previously described (Moreno et al. 2025). The intended constructions were subject to Sanger sequencing for confirmation, by Eurofins Genomics (Ebersberg, Germany).

### Plasmids

Supplemental Table S1 summarizes the plasmids employed in this work. Detailed plasmid maps and primers utilized in cloning steps are shown in Moreno et al. (2025). In-Fusion HD Cloning Kit (Takara Bio, Shiga, Japan) was used for plasmid assembly. The plasmids pCfB3035-Ply511_SEC, pCfB2904-Ply511_SEC, and pCfB2909-Ply511_SEC for integration and secretion of endolysin Ply511 were derived from the parental plasmid pCfB3035, pCfB2904, or pCfB2909 (Jessop-Fabre et al. 2016; Moreno et al. 2025) for targeted genomic integration into chromosomes X-4, XI-3, and XII-5, respectively, of *S. cerevisiae* or *S. boulardii*.

### Yeast strains and construction of recombinant* S. boulardii*

The yeast *S. cerevisiae* CEN.PK113-7D (Nijkamp et al. 2012) or *S. boulardii* CNCM I-745 (UL250, Biocodex, Gentilly, France) was used as a host for genetic transformation and is hereafter referred to as Scv or Sb wild-type (WT), respectively.

Transformations were performed using the polyethylene glycol/lithium acetate method described before (Gietz and Schiestl 2007), as described in Moreno et al. (2025).

Yeast cultures were grown and propagated at 30 °C for *S. cerevisiae* and 37 °C for *S. boulardii* and stored at 4 °C on YPD agar plates (1% yeast extract, 2% peptone, 2% glucose, 2% agar). If yeast strains carried plasmids, YPD media were supplemented with antibiotics as previously described in Moreno et al. (2025). For *S. boulardii*, half of each antibiotic concentration was used to promote faster post-transformation recovery.

For colony PCR verification, individual colonies were picked as described in Moreno et al. (2025) using specific primers (Supplemental Table S2).

### *L. monocytogenes *strains

*L. monocytogenes* used for this study was strain CECT 5672 (Serovar 4b), which was obtained from Colección Española de Cultivos Tipo (CETC). Tryptic Soy Broth (TSB; Sigma-Aldrich, Burlington, MA, USA) either TSB supplemented with 1.5% agar or Oxford Selective Agar (Sigma-Aldrich, Burlington, MA, USA) was employed for *L. monocytogenes* cultures or enumeration, respectively.

### Evaluation of enzymatic activity

The enzymatic activity of the yeast-expressed endolysin was assessed using an assay based on peptidoglycan degradation as previously described (Moreno et al. 2025).

### Yeast growth kinetics

Yeast growth was measured using a 96-well microplate in an automated system for continuous optical density monitoring at 600 nm (OD₆₀₀). Overnight cultures grown in YPD broth at 30 °C, afterwards were diluted 1:100 into fresh medium, and 200 µL of this suspension was dispensed into each well. A ThermoFisher microplate reader (Waltham, MA, USA) was used for measurements. Plates were incubated at 37 °C with orbital shaking, while OD₆₀₀ values were recorded every 15 min to track biomass concentration. All measurements were performed in triplicate under both aerobic and anaerobic conditions, using a type A vinyl anaerobic chamber (Coy Laboratory Products, Grass Lake, MI, USA).

### Preparation of yeast extracts

Yeast extracts were prepared according to Moreno et al. (2025). Briefly, after a pre-inoculum in YPD medium, cells were diluted to an OD₆₀₀ of 0.1 in 20 mL of fresh YPD medium in 100 mL Erlenmeyer flasks and then incubated at 30 °C with agitation at 200 rpm for 96 h. Then, cells were harvested by centrifugation at 5000×*g* for 10 min and washed three times with Tris buffer (50 mM Tris, 200 mM NaCl, pH 8.0) to achieve a final concentration of approximately 5 × 10⁹ colony-forming units (CFU)/mL.

The sonicated yeast extract was generated using a Cole-Parmer Ultrasonic Processor (Cole-Parmer, Vernon Hills, IL, USA), in two 10-min cycles consisting of alternating 30-s ON and OFF intervals at 40% amplitude. The resulting lysate was centrifuged at 10,000×*g* for 10 min to remove cellular debris, and the clarified supernatant was sterilized by filtration through a 0.22-µm membrane (MilliporeSigma, Burlington, MA, USA). The sterile extracts were either stored at 4 °C for immediate use or kept at −20 °C for later experiments.

### Anti-*Listeria* effect of yeast extracts on the SIHUMI Consortium

The SIHUMI consortium consists of a set of fully sequenced human-derived intestinal bacteria (Wohlgemuth et al. 2011). In this work, we used a modified version, which we named SIHUMI-L, since it includes *L. monocytogenes* CECT 5672. *E. coli *LF82*, Enterococcus faecalis* OG1RF,* Lactiplantibacillus plantarum* WCFS1,* Faecalibacterium duncaniae A2–165*,* Bifidobacterium longum* ATCC 15707,* Phocaeicola vulgatus* DSM1447, and *Mediterraneibacter gnavus *(previously termed *Ruminococcus gnavus*)ATCC 29149 (Togo et al. 2018) were grown in solid and liquid LYHBHI medium at 37 °C in strict anaerobic conditions and were maintained as single-use glycerol stocks at − 80 °C for long periods, as previously described (Sokol et al. 2008; Ríos Colombo et al. 2023, 2025).

We followed the protocol described previously (Ríos Colombo et al. 2023); briefly, each strain was grown individually for 24 h in 5 mL of LYHBHI at 37 °C under strict anaerobic conditions. Then, each strain was diluted as needed with LYHBHI to a final working OD_600_ of 1. Four different tubes (one per condition tested) were inoculated with 10 µl of each culture in 10 mL of LYHBHI, forming four initial SIHUMI consortia. To form the SIHUMI-L, at time 0, *L. monocytogenes* was added at a final concentration of 5 × 10^3^, simulating the dose needed for infection. Tubes were then incubated at 37 °C, statically, under anaerobic conditions. Also at time 0, simultaneously, yeast extracts (4 mL) were added to the different SIHUMI-L-containing tubes at time 0 h.

At 0, 6, 24, and 48 h after inoculation, 1 mL samples were collected. Cultures were immediately centrifuged at 10,000×*g* for 2 min to separate the supernatant from the cell pellets. Both fractions were collected and stored at − 20 °C until further analysis. In parallel, viable *L. monocytogenes* cells were quantified by plating serial dilutions of the SIHUMI-L culture onto Oxford Selective Agar (Sigma-Aldrich, Burlington, MA, USA). Plates were incubated at 37 °C for 24–48 h, after which colony-forming units (CFU/mL) were enumerated.

Total genomic DNA (gDNA) was extracted from bacterial pellets using the GenElute™ Bacterial Genomic DNA Kit (Sigma-Aldrich, Arklow, Ireland), following the manufacturer’s instructions. DNA was eluted in 200 µL of the provided elution buffer for each sample.

### Quantitative real-time PCR

To determine the genome copy number of each bacterial species in the SIHUMI-L consortium at the different sampling time points, quantitative PCR (qPCR) was employed as previously described (Ríos Colombo et al. 2023, 2025; Lawley et al. 2017; Lengfelder et al. 2019; Guerin et al. 2021), using species-specific primers listed in Supplemental Table S3, based on the total genomic DNA extracted from each sample.

### In vitro gastrointestinal digestion assay

In vitro simulation of gastric and small intestinal digestion was performed according to the INFOGEST protocol (Brodkorb et al. 2019), adapted to use 5 × 10^8^ CFU/mL of yeast (*S. boulardii* or *S. cerevisiae*) and 5 × 10^3^ CFU/mL of *L. monocytogenes* in 5 mL of whole milk, in duplicate.

Recombinant or wild-type yeast cultures were first grown overnight in YPD broth and subsequently diluted to an initial optical density (OD₆₀₀) of 0.1 in 20 mL of fresh YPD medium within 100 mL Erlenmeyer flasks. Cultures were incubated at 30 °C with agitation at 200 rpm for 96 h, reaching an approximate final density of 10⁹ CFU/mL. The viability of *L. monocytogenes* was determined by plating serial dilutions on Oxford Selective Agar (Sigma-Aldrich, Burlington, MA, USA). Plates were incubated at 37 °C for 24–48 h, after which CFU/mL values were calculated. Sampling was performed at three time points: the beginning of the experiment (0 h), following simulated gastric digestion (2 h), and after simulated intestinal digestion (22 h). Chemicals required were obtained from Sigma-Aldrich (St. Louis, MO, USA). We did not simulate the oral phase since the use of the yeast or its extracts would only imply swallowing and not mastication.

### In vitro intestinal fermentation with human fecal microbiota

Fecal samples were donated by two men and one woman comprising ages between 32 and 50 years and were recruited through a trial authorized by the Regional Ethics Committee for Clinical Research (Galician Health Service, SERGAS, n° 2018/270). Participants were selected according to the eligibility criteria of that trial, which required that volunteers had no gastrointestinal disorders, were not undergoing chronic medication, and had not taken antibiotics or pre-, pro-, or postbiotic supplements during the six months preceding sample collection. All participants provided written informed consent after being informed about the intended use of their biological material. In vitro simulation of human intestinal digestion was performed as previously described (López-Santamarina et al. 2025). To evaluate the potential use of modified and unmodified yeast as probiotics and its impact in the microbiota in the presence of a pathogen, a negative control with a pathogen and without yeast was carried out simultaneously. Conditions resembling those of the human small intestine, including a microaerophilic atmosphere and a temperature of 37 °C, were replicated using GENbox microaer Sachets (BioMerieux, Marcy-l'Étoile, France). The sterilized medium (composition indicated below) was mixed with 0.01% (v/v) of the previously diluted feces, and a final concentration of ~ 5 × 10^4^ CFU/mL of *L. monocytogenes* and ~ 5 × 10^6^ of *S. boulardii* or *S. cerevisiae* was inoculated into the vessels. The assays were performed for 48 h; 2 mL samples were taken for analysis at 0, 6, 24, 30, and 48 h of fermentation. The total volume was 10 mL.

Viable *L. monocytogenes* was quantified by plating serial dilutions onto Oxford Selective Agar (Sigma Aldrich, Burlington, MA, USA), followed by incubation at 37 °C for 24 h or 48 h, to count and calculate CFU/mL. The estimation of yeast CFUs at different time points (0, 24, and 48 h) was determined from the total gDNA extracted, by qPCR using specific primers targeting the genomic modifications in engineered *S. cerevisiae* or *S. boulardii* secreting Ply511 (Forward: AGACAAGCTGGCCAAACAGT, Reverse: CTGGTGCTTTGTTTGTGGGG) or primers targeting *Saccharomyces* spp*.* (Chang et al. 2007) (Forward: AGGAGTGCGGTTCTTTG, Reverse: TACTTACCGAGGCAAGCTACA). A medium was prepared according to previous studies (Cueva et al. 2015) with slight modifications in its composition: arabinogalactan (1 g/L), pectin from apple (2 g/L), inulin (1 g/L), potato starch (3 g/L), glucose (0.4 g/L), yeast extract (3 g/L), peptone (1 g/L), mucin (4 g/L), and L-cysteine (0.5 g/L). All compounds were dissolved in 1 L of distilled water and sterilized at 121 °C for 15 min.

### Statistics

All data analysis was performed in GraphPad Prism (version 9.0.0; GraphPad, San Diego, CA, USA). Unpaired *t*-test was used to evaluate statistical significance. The upper threshold for statistical significance for all experiments was set at *p* < 0.05.

## Results

### *S. boulardii *triple integration of the Ply511 secretion cassette

Previously, we implemented that a CRISPR-Cas9 strategy, which allowed us to engineer *S. cerevisiae* to secrete the anti-*Listeria* endolysin Ply511 and reduce levels of *L. monocytogenes* (Moreno et al. 2025). Given that *S. boulardii* is a probiotic yeast already used commercially, we aimed to introduce the same genetic modification into its genome to enhance its antibacterial activity and broaden its potential health benefits.

**Fig. 1:**
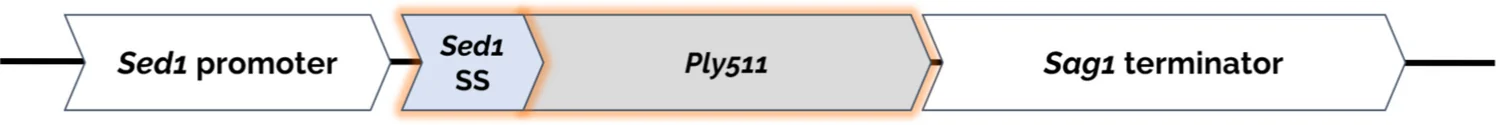
Endolysin secretion cassette integrated into *S. boulardii*. The coding sequence is highlighted with orange (Sed1 secretion signal-Ply511), preceded by the *Sed1* promoter and followed by the *Sag1* terminator

To allow the secretion of Ply511, we used the secretion cassette used in Moreno et al. (2025), which contained the DNA sequences for the *Sed1* promoter, the Sed1 secretion signal peptide, the Ply511 endolysin, and the *Sag1* terminator (Fig. 1). Via CRISPR-Cas9, we integrated the endolysin-expressing cassette into three loci on chromosomes X, XI, and XII (Supplemental Fig. S1) creating the strain *Sb-Ply511-X-4-XI-3-XII-5*.

### *S. boulardii *secreting Ply511 shows enzymatic activity

**Fig. 2:**
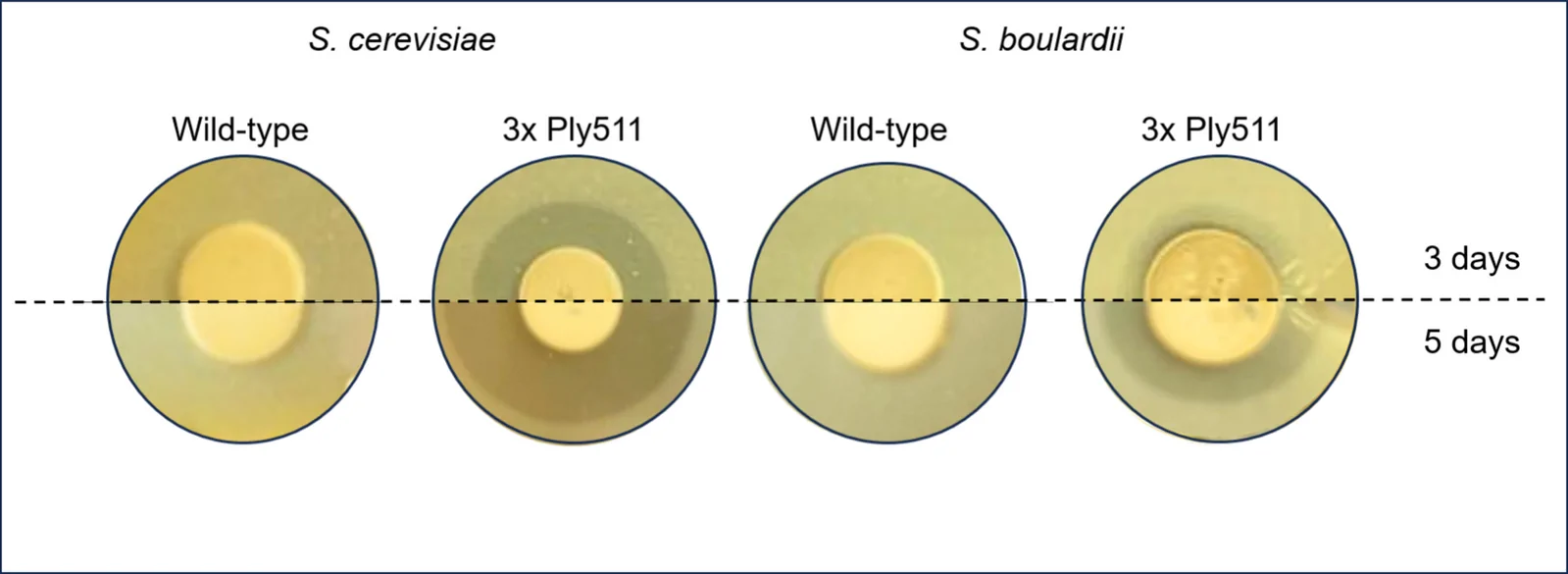
Enzymatic activity of *S. cerevisiae* or *S. boulardii* displaying endolysin Ply511 or wild type over a heat-killed layer of *L. monocytogenes* Scott A

To confirm the muralytic activity of *S. boulardii* expressing Ply511, we first tested the yeast’s ability to degrade peptidoglycan. Each yeast was spotted on an opaque YPD-agar mixture containing heat-killed cells of *L. monocytogenes*. We observed that after 48 h of incubation, the recombinant yeast was able to degrade *L. monocytogenes* peptidoglycan layer, by indication of clearance around its colonies, similar to the activity of *S. cerevisiae* previously reported (Moreno et al. 2025). In contrast, no clearance was observed around the wild-type yeast spots (Fig. 2). Although this assay is qualitative (positive or negative) and not quantitative, we did observe a difference in halo diameter between both engineered yeasts over the course of 3 to 5 days (Fig. 2), which could indicate a higher secretion rate in *S. cerevisiae* than in *S. boulardii*.

### Growth of *S. boulardii* secreting Ply511 differs depending on oxygen levels

*S. boulardii* secreting Ply511 showed no growth impairment aerobically and reached a similar growth (OD_600_) to its wild-type counterpart over a period of 24 h (Supplemental Fig. S2). When its growth was tested anaerobically, *S. boulardii* secreting Ply511 did not achieve the same optical density as the wild type; after 24 h, the OD_600_ of the engineered yeast was 0.795, in comparison to the OD_600_ of 0.999 for the wild type. This difference was not observed in *S. cerevisiae* (Supplemental Fig. S2).

### Yeast extracts inhibit *Listeria* in the SIHUMI community

We previously reported (Moreno et al. 2025) that among the tested forms, yeast cells secreting endolysin and corresponding cell extracts, the cell extracts exhibited higher anti-*Listeria* activity in *S. cerevisiae* secreting the endolysin. However, it has been shown that the microbial context in which an antimicrobial acts can influence its activity (Bottery et al. 2021). Thus, to assess whether this activity would be replicated in a polymicrobial community of intestinal bacteria that would mimic intestinal conditions in a controlled manner, we used our own version of the SIHUMI community (SIHUMI-L), composed of *E. coli*, *E. faecalis*,* F. duncaniae*,* B. longum*,* P. vulgatus*, *M. gnavus*, and *L. monocytogenes.*

We added different yeast extracts to SIHUMI-L (*S. boulardii* or *S. cerevisiae* wild-type or *S. cerevisiae* and *S. boulardii* secreting Ply511) together with a non-yeast control (containing only the SIHUMI-L community without any yeast extract).

**Fig. 3:**
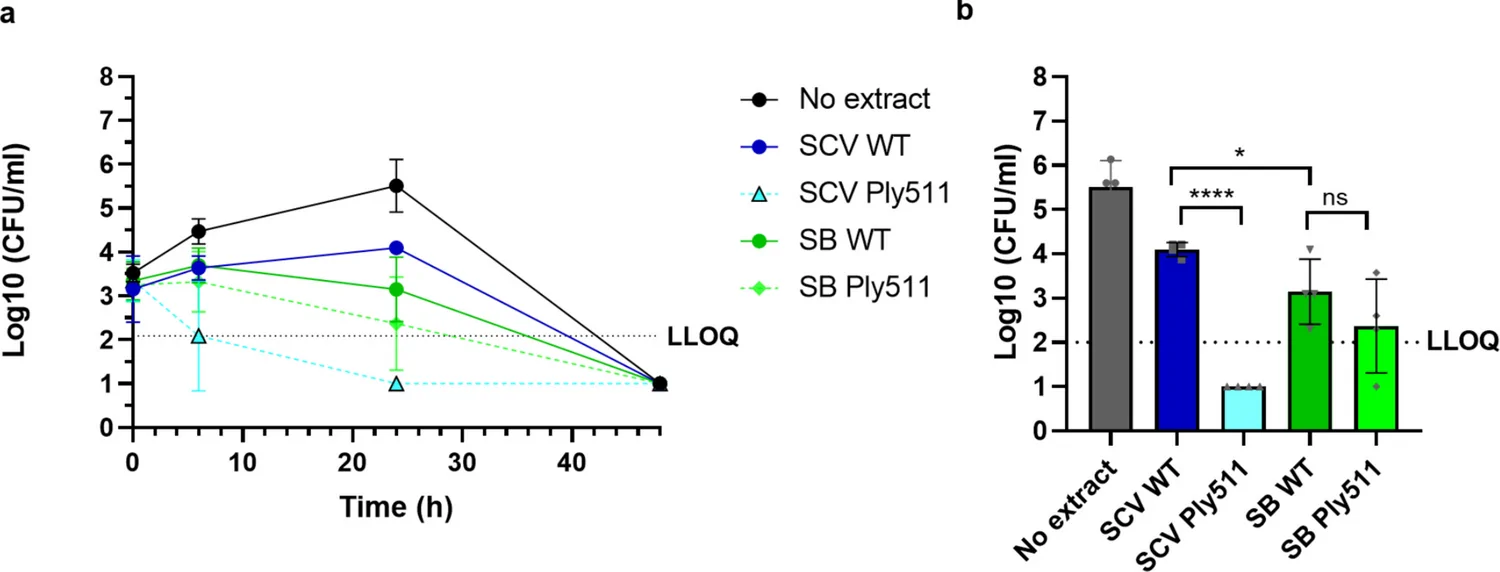
**a** Log10 (CFU/mL) of *L. monocytogenes* over time, upon mixture with the SIHUMI consortium (black), plus *S. cerevisiae* cell extract wild-type (WT) (dark blue), *S. cerevisiae* secreting Ply511 (light blue) or *S. boulardii* cell extract WT (dark green), and *S. cerevisiae* secreting Ply511 (light green) for a period of 48 h. **b** Log10 (CFU/mL) of *L. monocytogenes* upon mixture with the SIHUMI consortium (black), plus *S. cerevisiae* cell extract WT (dark blue), *S. cerevisiae* secreting Ply511 (light blue) or *S. boulardii* cell extract WT (dark green), and *S. cerevisiae* secreting Ply511 (light green) at 24 h

We found that *L. monocytogenes* was able to grow within the SIHUMI consortium as indicated in Fig. 3a (no extract condition) during the first 24 h, then fell after 48 h to levels below the limit of quantification. When in contact with all of the yeast extracts, *L. monocytogenes* levels were lower. The extract from *S. cerevisiae* Ply511 showed the highest anti-*Listeria* activity, reducing *L. monocytogenes* by 1.6 Log10 (CFU) at 6 h in comparison with the wild-type extract, and could not be detected after 24 h (Fig. 3b). The extract from *S. boulardii* containing Ply511 only led to a slight (non-statistically significant) reduction of *L. monocytogenes* after 24 h (Fig. 3b). These differences in activity between *S. cerevisiae* and *S. boulardii* are in accordance with those observed in Fig. 3.

### Yeast extracts do not affect the members of the SIHUMI community

**Fig. 4:**
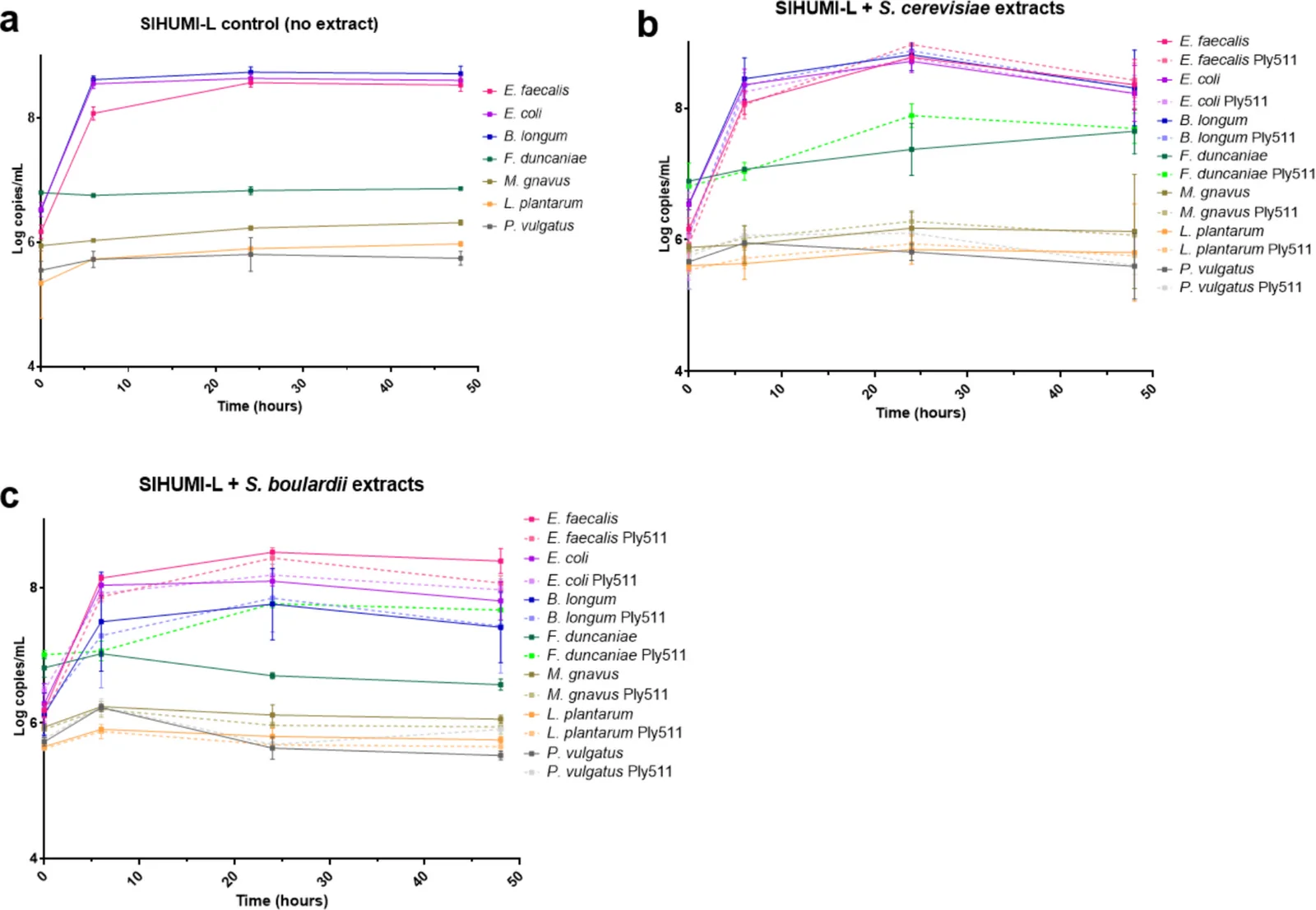
Genome copies/mL over time (0, 6, 24, and 48 h) of members of the SIHUMI consortium in LYHBHI after inoculation with *L. monocytogenes* at time 0. Each time point is represented as a mean with standard deviation of 4 replicates. **a** SIHUMI-L control (no yeast extract). **b** SIHUMI-L with added *S. cerevisiae* extracts containing either Ply511 or the wild-type counterpart or **c** SIHUMI-L with added *S. boulardii* extracts containing either Ply511 or the wild-type counterpart

Next, we evaluated the impact of this treatment on the rest of the members of the SIHUMI-L community by qPCR. No difference was observed in the outcome of the treatments with the yeast extract with or without the endolysin, which indicates that the treatment does not affect the other six bacterial species present in the community. *E. faecalis*, *E. coli*, and *B. longum* grew after 6 h and dominated throughout the 48 h tested. *F. duncaniae* grew slightly when mixed with *S. cerevisiae* extracts (Fig. 4b), regardless of whether those were producing Ply511 or not. When mixed with *S. boulardii* producing the endolysin (Fig. 4c), *F. duncaniae* was able to grow, but not in the presence of the extract of the wild-type *S. boulardii. M. gnavus* and *P. vulgatus* were unable to grow in the SIHUMI-L community, with or without extracts. This can be the result of its interactions with *L*. *monocytogenes* or can be due to the cells not being viable, as depicted in Fig. 4 Also, *E. faecalis,* another member of the community has been reported to inhibit these strains, resulting in little to no growth in the consortium (Ríos Colombo et al. 2023, 2025)

### *S. cerevisiae *and *S. boulardii *secreting Ply511 are active against* L. monocytogenes* in simulated gastric and intestinal fluids

After confirming the anti-*Listeria* activity of the yeast extracts, we hypothesized that yeast should better resist gastrointestinal conditions than their extracts and tested both *S. cerevisiae* and *S. boulardii* whole cells.

**Fig. 5:**
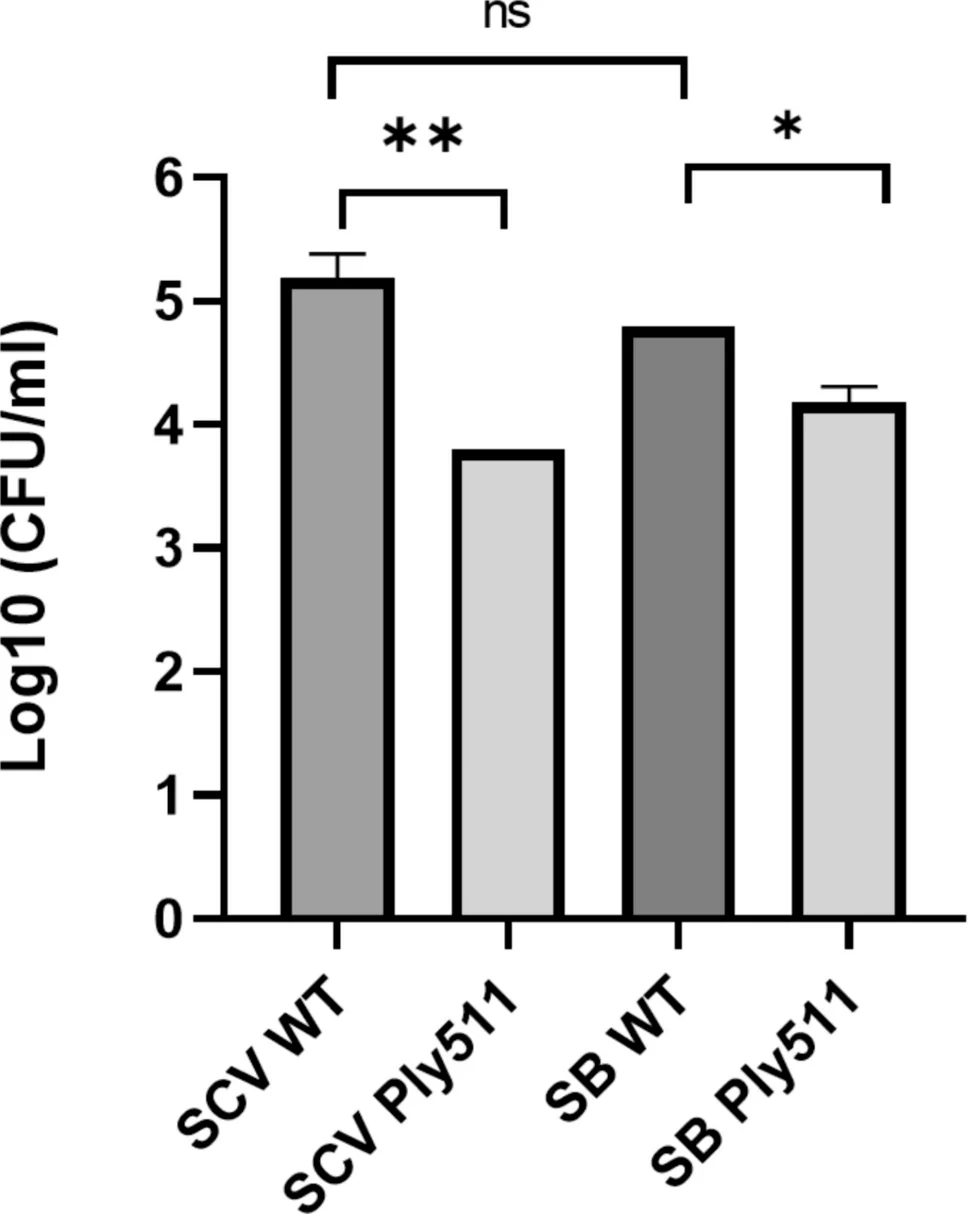
Log_10_ (CFU/mL) of *L. monocytogenes* upon mixture with *S. boulardii* (SB) *or S. cerevisiae* (SCV), wild-type (WT) or secreting Ply511 (Ply511), as indicated in the legend, after 24 h of contact (2 h in SGF and 22 h in SIF). The figure shows the Log_10_ (CFU/mL) mean of three replicates; standard deviation is represented as error bars. The unpaired *t* test comparison between the WT yeast and the yeast secreting Ply511 at 24 h is indicated as follows: ****p ≤ 0.0001, ***p ≤ 0.001, **p ≤ 0.01, *p ≤ 0.05 and ns, not significant *p* ≥ 0.05

First, to assess if the recombinant *S. cerevisiae* or *S. boulardii* could effectively reduce *L. monocytogenes* serovar 4b in conditions resembling gastrointestinal ingestion, we mixed milk and bacterial and yeast cells in simulated gastric fluid (SGF) at pH 3 for 2 h and then for 24 extra hours in simulated intestinal fluid at pH 7. We monitored viable *L. monocytogenes* cells over time. As shown in Fig. 5, both recombinant *S. cerevisiae* and *S. boulardii* secreting Ply511 significantly reduced *L. monocytogenes* levels after 24 h compared with their wild-type counterpart. *S. cerevisiae* secreting Ply511 reduced *Listeria* levels by 1.39 ± 0.19 Log_10_ (CFU/mL) in comparison with *S. cerevisiae* wild-type and *S. boulardii* secreting Ply511 reduced *Listeria* levels by 0.61 ± 0.12 Log_10_ in comparison with *S. boulardii* wild-type.

### *S. boulardii *reduces *L. monocytogenes* during in vitro intestinal fermentation

Next, we wanted to test if recombinant yeast were able to grow and selectively reduce *L. monocytogenes* during intestinal fermentation, considering the competition against the intestinal microbiota of three human donors. We mixed the fecal samples with *L. monocytogenes* and the different yeasts (*S. boulardii* or *S. cerevisiae* wild-type or *S. cerevisiae* and *S. boulardii* secreting Ply511) and a non-yeast control (containing only the fecal microbiota).

**Fig. 6:**
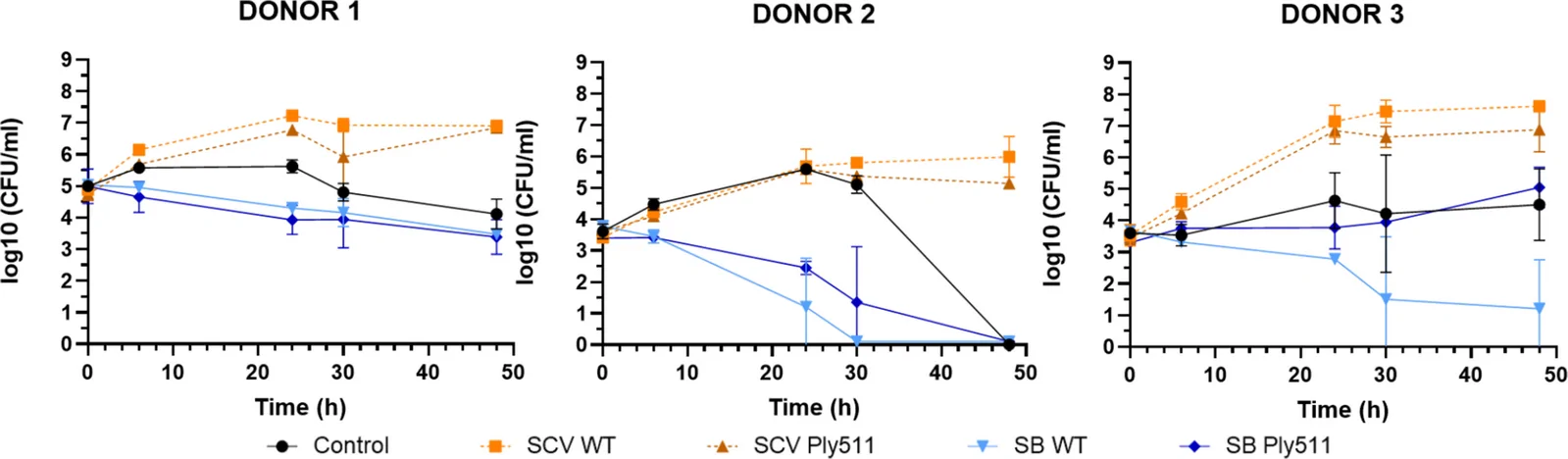
Log_10_ (CFU/mL) of *L. monocytogenes* serovar 4b upon mixture with *S. boulardii *(SB)* or S. cerevisiae* (SCV), wild-type (WT) or secreting Ply511 (Ply511), as indicated in the legend, during 48 h of contact with human feces from 3 different donors in anaerobic conditions. The figure shows the Log_10_ (CFU/mL) mean of two replicates; standard deviation is represented as error bars

We observed a similar trend in all 3 donors (Fig. 6):

First, when no yeast was used, *L. monocytogenes* grew in the first 24 h and decreased slightly in the 24 h to 48 h period. *L. monocytogenes* numbers were not detected after 48 h only in 1 out of 3 donors, suggesting that this specific microbiome is more hostile towards *Listeria*.

Second, *L. monocytogenes* numbers grew over time when using either wild-type or recombinant *S. cerevisiae.* We observed in all three cases that the recombinant *S. cerevisiae* performed slightly better than the wild-type and reduced *L. monocytogenes* at 30 and 48 h, in comparison to the wild type. The concentration of *Listeria* was, however, higher in both cases in comparison to the control that did not contain yeast, suggesting that *L. monocytogenes* growth was favored when introducing *S. cerevisiae* in the intestinal conditions.

Third, *S. boulardii* reduced *L. monocytogenes* at all tested time points compared to the control group (except for donor 3, time 48 h). *S. boulardii* wild type showed a similar trend as the recombinant *S. boulardii* expressing the endolysin Ply511. However, *L. monocytogenes* numbers were generally slightly lower in the wild-type yeast. In fact, in 2 out of 3 donors, *S. boulardii* wild-type treatment was able to reduce *L. monocytogenes* below the limit of detection. The recombinant *S. boulardii* was able to reduce *L. monocytogenes* below the limit of detection in 1 out of 3 donors.

Yeast growth during the 48 h fermentation was estimated via qPCR with specific primers for each strain. Both wild type and recombinant strains were detected throughout the 48 h (Supplemental Fig. S3). Estimated CFUs/mL were increased over time in all cases, as shown in Supplemental Fig. S3, indicating that both *S. cerevisiae* and *S. boulardii* were metabolically active and growing during the tested conditions.

## Discussion

In this study, we show that engineered *S. boulardii* and *S. cerevisiae* can secrete endolysin Ply511 with activity against *L. monocytogenes*, highlighting their potential for delivery in the gut.

Firstly, we used CRISPR-Cas9 to engineer *S. boulardii* with a scarless triple chromosomal integration free of antibiotic resistance markers and demonstrated that it can express an enzymatically active protein. The avoidance of antibiotic markers is essential in terms of regulations governing any potential therapeutic application of genetically modified organisms. When comparing the *S. boulardii* strain to our previously engineered *S. cerevisiae* strain carrying the same triple chromosomal integration (Moreno et al. 2025), we observed lower antibacterial activity in most assays. This reduced activity is likely due to the fact that the expression cassette and secretion signal were originally optimized for *S. cerevisiae* (Inokuma et al. 2016) and may require further adaptation to maximize expression and/or secretion in *S. boulardii* commercial strains. In fact, in our enzymatic assays, *S. cerevisiae* showed higher activity, indicating a higher endolysin expression. This indication remains to be confirmed by quantitative assays. While we are comparing two closely related species, it is important to note that even within a single species*, S. cerevisiae*, the same genetic modification can lead to different outcomes depending on the strain background, such as laboratory versus industrial strains (Costa et al. 2021, 2022). This strain-dependent variability supports our observation that the same engineering strategy may not yield identical results in *S. boulardii* and *S. cerevisiae*, highlighting the importance of host-specific optimization. An example of this is the lower protein display efficiency in *S. boulardii* vs *S. cerevisiae* (Xu et al. 2025). In addition, differences in glycosylation patterns between *S. cerevisiae* and *S. boulardii* may further influence the functionality of the expressed protein.

Despite differences in activity, there are several advantages of using the probiotic *S. boulardii* for the delivery of endolysins over *S. cerevisiae*, despite being more challenging to engineer (Chen et al. 2020). First, it is already marketed as a probiotic and shows phenotypic traits that make it suitable for better survival in the GI tract, namely its high tolerance to acidic conditions and its ability to grow well at 37 °C, human core temperature (Pais et al. 2020). *S. boulardii* has also shown anti-bacterial effects and is known to help maintain the integrity of the intestinal mucosa (Carvalho et al. 2025) and is already used in the clinic to reduce antibiotic side effects such as dysbiosis (Lau and Chamberlain 2016). Moreover, the probiotic has been shown to be suitable for transient delivery of molecules (Hedin et al. 2023) since its residence times in the gut are low (Blehaut et al. 1989; Elmer et al. 1999). Finally, these probiotic traits could serve to potentiate the antibacterial activity of endolysins.

We previously found engineered yeast cell extracts to have the highest activity against *L. monocytogenes*, compared to whole cells and supernatants; thus, we tested both *S. cerevisiae* and *S. boulardii* extracts against *L. monocytogenes* in a controlled simplified microbiome (SIHUMI-L) under anaerobic conditions. The extracts showed an effective anti-*Listeria* activity. When we evaluated the effect of the endolysin on other members of the SIHUMI community, we found that it is specific to *Listeria*, as it did not affect the survival of the other members compared to a consortium control with no extract. The remaining four members of the community, *R. gnavus*,* F. prausnitzii*,* L. plantarum*, and *P. vulgatus*, were unable to grow in the presence of *L. monocytogenes*, even without the addition of yeast extract. This lack of growth may be due to antagonistic interactions (such as those posed by *E. faecalis)* or due to culture viability issues, but not due to the endolysin treatment itself. *E. faecalis* and *B. longum* are among the dominant bacteria in the consortium. These are Gram-positive bacteria and could, in principle, be more prone to endolysin attack (unlike Gram-negative species such as *E. coli* or *P. vulgatus*). However, their growth was not affected by either of the cell extracts tested, which can be explained by the different peptidoglycan chemotype of these bacteria compared to *Listeria,* therefore not being targeted by Ply511. The cell-binding domain of Ply511 has a broad binding range and has been shown to bind all *Listeria* serovars (Schmelcher et al. 2010). The same has been observed with other species; for example, the cell-binding domains of other anti-*Listeria* endolysins have shown affinity to *Bifidobacterium* strains, suggesting some degree of similarity in their cell wall structures. However, the fact that *B. longum* was able to grow in the presence of our endolysin-containing extracts highlights the specificity of this treatment, reinforcing the potential of Ply511 as a microbiome-sparing therapeutic agent.

Since listeriosis can be fatal, particularly in vulnerable populations, we could envision the use of these yeast extracts as processing aids for bio-preservation of certain food products prone to *Listeria* contamination. This approach could be especially valuable for fermented foods such as cheese, yogurts, and other products where a selective anti-*Listeria* treatment would be beneficial if compatible with yeast fermentation processes. An effective application likely depends on achieving sufficient endolysin activity and maintaining protein stability across variable pH, temperature, and proteolytic conditions found in food matrices or the GI tract; such as the previously observed activity of Ply511-containing extracts in milk (Moreno et al. 2025) or the anti-*Listeria* activity of yeast cells that we report upon the simulated in vitro digestion. Beyond bio-preservation, another potential application can be as engineered live microorganisms, which could be used as adjunctive therapy to antibiotics or prophylactically against *L. monocytogenes*. From a regulatory perspective, postbiotics such as supernatants or yeast extracts may offer a more straightforward pathway than live genetically modified probiotics, since only two of such products are marketed to date (Carvalho et al. 2025).

To test if yeast could serve as a delivery vehicle for endolysin in the gut, we simulated gastrointestinal digestion by co-incubating *L. monocytogenes* with the engineered yeasts in milk. After 24 h in simulated intestinal fluid, *Listeria* counts were reduced by 1.4 Log units with *S. cerevisiae* and 0.6 Log units with *S. boulardii*, corresponding to a 96% or 75% reduction, respectively, compared to their wild-type counterparts. These results suggest a positive antimicrobial effect and highlight the potential of this approach for the treatment or prevention of *Listeria* infections. To better understand the interactions within the gut environment, we simulated an intestinal fermentation by co-incubating *S. cerevisiae* or *S. boulardii* (wild-type or engineered) with a human fecal sample and inoculating *L. monocytogenes* under conditions mimicking the small intestine, where *Listeria* translocation normally occurs (Nikitas et al. 2011), over a 48-h period. Under these conditions, *S. boulardii* was effective in reducing the bacterial load, whereas *S. cerevisiae* appeared to promote *Listeria* growth, possibly serving as a nutrient source and resulting in higher bacterial counts. We observed a similar phenomenon during our previous work (Moreno et al. 2025), where *S. cerevisiae* wild type seemed to promote *L. monocytogenes* growth. We do not observe this effect for *S. boulardii*, and our assay with the SIHUMI consortium seems to indicate that the other bacteria of the community did not benefit from this growth. This suggests that this is a *Listeria*-specific effect rather than a limitation of yeast-based delivery systems. While the exact reason remains unknown, this growth is likely to stem from the release of certain nutrients from the metabolically active *S. cerevisiae* or due to the yeast providing an attachment surface for cells*.*

However, we did not observe a significant difference in *L. monocytogenes* reduction between the engineered and wild-type yeast strains, suggesting that endolysin secretion did not enhance the antibacterial effect under these conditions. This limited activity may be due to the competition between the yeast and the complex microbiota present in the fecal samples, potentially resulting in reduced yeast metabolic activity and, consequently, low expression of Ply511, insufficient to effectively suppress *L. monocytogenes*. These findings also underscore the contrast between testing treatments in simplified microbial communities versus in human fecal samples, the latter presenting a far more complex and diverse environment. To gain a deeper insight into which microbial populations are affected and how the different yeast strains exert their effects, metagenomic sequencing should be performed.

Yeast metabolic activity is also affected by the lack of oxygen and low nutrient availability, which likely drives the activation of yeast stress response, something that has been shown to affect constitutive promoter strength (Xiong et al. 2018) and might be a limitation for expression under gut-like conditions. Expression systems specifically designed for such environments, like the use of stronger promoters (Sands et al. 2024) or stress-responsive induction promoters (Xiong et al. 2018), could be of interest to overcome this limitation. Future efforts may benefit from a modular and systematic approach, such as the described GoldenGate (Agmon et al. 2015) or more specifically VersaTile (Gerstmans et al. 2020) strategy, where combinatorial libraries containing different secretion signals, promoters, endolysin coding sequences, and terminators can be tested and optimized while being assembled into a final expression system. These approaches could be optimized to include selection filters such as conditions of low pH, low nutrient availability, or low-oxygen conditions resembling the GI tract.

Recently, Cho et al. (2024) studied the effect of endolysin CD27L_EAD in the gut microbiome, demonstrating the specific reduction of *Clostridioides difficile* and showing differences between vancomycin and endolysin treatment. Endolysins have shown promising results in other microbiome-relevant contexts, such as the skin and vaginal tract (Landlinger et al. 2021; Wilkinson et al. 2024). While more data are needed to conclude their microbiome-sparing effect, a growing body of evidence supports this potential.

This study demonstrates the capacity of yeast as a vehicle for endolysin delivery. With further improvements, this approach could lead to a platform of probiotics suitable for endolysin delivery against intestinal pathogens leading to the prevention of certain infections or targeted interventions in the microbiome.

## Supplementary Information

Below is the link to the electronic supplementary material.

ESM1 (DOCX 784 KB)

## Data Availability

Data is provided within the manuscript or supplementary information files.

## Declarations

### Declaration of generative artificial intelligence and artificial intelligence-assisted technologies in the writing process

During the preparation of this work, the authors used ChatGPT to improve clarity and correct grammatical errors. All content was subsequently reviewed and edited by the authors, who take full responsibility for the final publication.
