# Supplementary material for: Evaluation of the delivery of an anti-Listeria endolysin via CRISPR-Cas9 engineered probiotic Saccharomyces boulardii
Source: Appl Microbiol Biotechnol. 2026 Feb 26;110(1):81. doi: 10.1007/s00253-026-13749-6 (PMC12948783; doi:10.1007/s00253-026-13749-6)
Supplement: Supplementary file 1 — (DOCX 784 KB) [file 253_2026_13749_MOESM1_ESM.docx]

SUPPLEMENTARY MATERIAL.

APPLIED MICROBIOLOGY AND BIOTECHNOLOGY.

Evaluation of the delivery of an anti-*Listeria* endolysin via CRISPR-Cas9 engineered probiotic *Saccharomyces boulardii*

David Sáez Moreno^1,^ , Joao Paulo Carvalho^1^, Ellen Murray^3^, Natalia Soledad Ríos Colombo^3^, Alexandre Lamas^4^, Alejandra Cardelle Cobas^4^, Colin Hill^3^, Joana Azeredo^1,2*^, Lucília Domingues^1,2*^

^1^ CEB - Centre of Biological Engineering, University of Minho, Braga, Portugal.

^2^ LABBELS - Associate Laboratory, Braga, Guimarães, Portugal.

^3^ APC Microbiome Ireland, University College Cork, Cork, Ireland

^4^ University of Santiago De Compostela, Lugo, Spain.

^*^ Correspondence to: [jazeredo@deb.uminho.pt](mailto:jazeredo@deb.uminho.pt), [luciliad@deb.uminho.pt](mailto:luciliad@deb.uminho.pt)

**Supplemental Materials:**

**
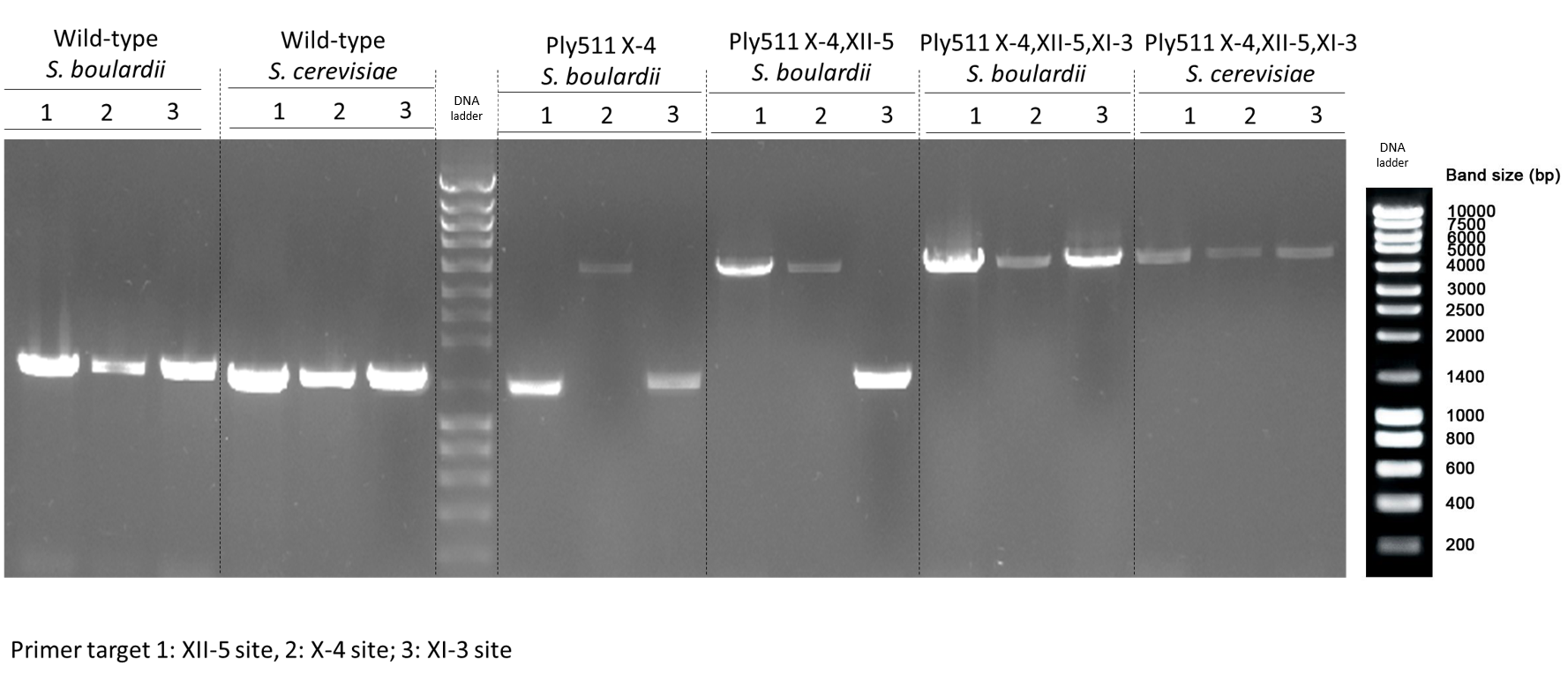
**

Supplemental Figure S1. Colony PCR of *S. boulardii* single, double and triple insertion of the Ply511 secretion cassette. The picture shows the gel electrophoresis 1% agarose of the PCR products, corresponding to the numbers indicated in the figure legend 1: Chromosome XII site 5 (primers 899 and 900); 2: Chromosome X site 4 (primers 905 and 906) 3: Chromosome XI site 3 (primers 911 and 912), LD: DNA ladder. Sizes expected for unmodified yeast, 1 (XII-5): 1365 bp, 2 (X-4): 1394 bp, 3 (XI-3) 1450 bp. For yeast secreting the endolysin, 1 (XII-5): 3799 bp, 2 (X-4): 3828bp, 3 (XI-3): 3884 bp.


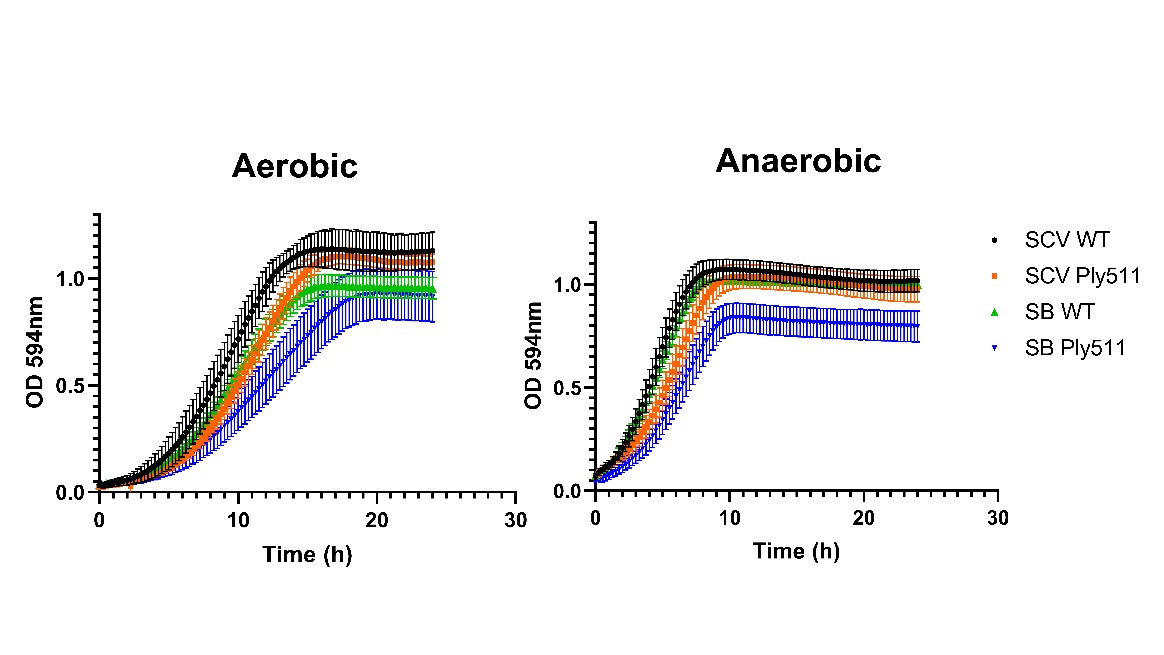


Supplemental Figure S2. OD600 measurements over 24 h, of different yeast over time, as indicated in the legend (SCV refers to *S. cerevisiae*, SB refers to *S. boulardii* and WT refers to wild-Type).

Supplemental Figure S3. Log10 (CFU/mL) of *S. boulardii* (SB) or *S. cerevisiae* (SCV), wild-type (WT) or secreting Ply511 (Ply511), as indicated in the legend, after 0 h, 24 h or 48 h of simulated intestinal fermentation. The figure shows the log10 (CFU/mL) mean of three replicates, standard deviation is represented as error bars.

Table S1. Plasmids used in this study.

| Plasmid name | Description | Integration site | Reference |
| --- | --- | --- | --- |
| pCfB3035-Ply511_SEC | Plasmid containg DNA sequences encoding for *SED1* promoter, *SED1*SS, Ply511, *SAG1* terminator and homology arms for site X-4 | X-4 | (Moreno et al. 2025) |
| pCfB2904-Ply511_SEC | Plasmid containing DNA sequences encoding for *SED1* promoter, *SED1*SS, Ply511, *SAG1* terminator and homology arms for site XI-3 | XI-3 | (Moreno et al. 2025) |
| pCfB2909-Ply511_SEC | Plasmid containing DNA sequences encoding for *SED1* promoter, *SED1*SS, Ply511, *SAG1* terminator and homology arms for site XII-5 | XII-5 | (Moreno et al. 2025) |
| pCfB2312 (Cas9) | Cas9 expression plasmid for marker-free integration | - | (Jessop-Fabre et al. 2016) |
| pCfB3042 (gRNA-X-4) | Guide RNA plasmid targeting site X-4 | X-4 | (Jessop-Fabre et al. 2016) |
| pCfB3045 (gRNA-XI-3) | Guide RNA plasmid targeting site XI-3 | XI-3 | (Jessop-Fabre et al. 2016) |
| pCfB3050 (gRNA-XII-5) | Guide RNA plasmid targeting site XII-5 | XII-5 | (Jessop-Fabre et al. 2016) |
|  |  |  |  |

Table S2. Primers employed in this work for the confirmation of the chromosomal insertion in *S. cerevisiae* or *S. boulardii*.

| Primer ID | Oligonucleotide sequence (5'–3') | Target locus | Reference |
| --- | --- | --- | --- |
| **905** | CTCACAAAGGGACGAATCCT | X-4 | (Jessop-Fabre et al. 2016) |
| **906** | GACGGTACGTTGACCAGAG | X-4 | (Jessop-Fabre et al. 2016) |
| **911** | GTGCTTGATTTGCGTCATTC | XI-3 | (Jessop-Fabre et al. 2016) |
| **912** | CACATTGAGCGAATGAAACG | XI-3 | (Jessop-Fabre et al. 2016) |
| **899** | CCACCGAAGTTGATTTGCTT | XII-5 | (Jessop-Fabre et al. 2016) |
| **900** | GTGGGAGTAAGGGATCCTGT | XII-5 | (Jessop-Fabre et al. 2016) |

Table S3. Selective species-specific primers used for quantification of SIHUMI consortium members.

| **Target strain** | **Genome size (bp)** | **Genome mass (ng)** | **Oligonucleotide sequence (5´ to 3´)** | **Amplicon size** | **Ref.** |
| --- | --- | --- | --- | --- | --- |
| *E. faecalis* OG1RF | 2739625 | 3.00 X 10^6^ | F: ACGGAGATTGTCACGCTTAGT  R: TCGGCATTATCTGGGTGGTC | 122 bp | (Buttimer et al. 2022) |
| *E. coli* LF82 | 4773108 | 5.23 X 10^6^ | F: CGGGTGTTGTCCTAACTGCT  R: CGAGTGGTCATTGGCCTCAT | 107 bp | (Buttimer et al. 2022) |
| *M. gnavus* ATCC 29149 | 3549191 | 3.89 X 10^6^ | F: GCGTGCTTGTATTCCGGATG  R: GCCTGAACAGTTGCTTTCGG | 115 bp | (Guerin et al. 2021) |
| *F. duncaniae* A2–165 | 3102523 | 3.40 X 10^6^ | F: TATTGCACAATGGGGGAAAC  R:CAACAGGAGTTTACAATCCGAAG | 77 bp | (Lengfelder et al. 2019) |
| *P. vulgatus* DSM1447 | 4773108 | 5.23 X 10^6^ | F: AAGCAGCAGGGAAATGTGGA  R: CTTTCCTTACTTGCGCGTCG | 142 bp | (Buttimer et al. 2022) |
| *L. plantarum* WCFS1 | 3308274 | 3.63 X 10^6^ | F: CGAAGAAGTGCATCGGAAAC  R: TCACCGCTACACATGGAGTT | 71 bp | (Lengfelder et al. 2019) |
| *B. longum* ATCC 15707 | 2385164 | 2.61 X 10^6^ | F: GAGGCGATGGTCTGGAAGTT  R: CCACATCGCCGAGAAGATTC | 108 bp | (Lawley et al. 2017) |

References

Buttimer C, Sutton T, Colom J, Murray E, Bettio PH, Smith L, Bolocan AS, Shkoporov A, Oka A, Liu B, Herzog JW, Sartor RB, Draper LA, Ross RP, Hill C (2022) Impact of a phage cocktail targeting *Escherichia coli* and *Enterococcus faecalis* as members of a gut bacterial consortium in vitro and in vivo. Front Microbiol 13:936083. https://doi.org/10.3389/FMICB.2022.936083

Guerin E, Shkoporov AN, Stockdale SR, Comas JC, Khokhlova E V., Clooney AG, Daly KM, Draper LA, Stephens N, Scholz D, Ross RP, Hill C (2021) Isolation and characterisation of ΦcrAss002, a crAss-like phage from the human gut that infects *Bacteroides xylanisolvens*. Microbiome 9. https://doi.org/10.1186/S40168-021-01036-7

Jessop-Fabre MM, Jakočiūnas T, Stovicek V, Dai Z, Jensen MK, Keasling JD, Borodina I (2016) EasyClone-MarkerFree: A vector toolkit for marker-less integration of genes into *Saccharomyces cerevisiae* via CRISPR-Cas9. Biotechnol J 11:1110–1117. https://doi.org/10.1002/BIOT.201600147,

Lawley B, Munro K, Hughes A, Hodgkinson AJ, Prosser CG, Lowry D, Zhou SJ, Makrides M, Gibson RA, Lay C, Chew C, Lee PS, Wong KH, Tannock GW (2017) Differentiation of *Bifidobacterium longum* subspecies longum and infantis by quantitative PCR using functional gene targets. PeerJ 2017:e3375. https://doi.org/10.7717/PEERJ.3375

Lengfelder I, Sava IG, Hansen JJ, Kleigrewe K, Herzog J, Neuhaus K, Hofmann T, Sartor RB, Haller D (2019) Complex bacterial consortia reprogram the colitogenic activity of *Enterococcus faecalis* in a gnotobiotic mouse model of chronic, immune-mediated colitis. Front Immunol 10:3389. https://doi.org/10.3389/FIMMU.2019.01420

Moreno DS, Cunha J, de Melo LDR, Tanaka K, Bamba T, Hasunuma T, Azeredo J, Domingues L (2025) CRISPR-Cas9 engineered *Saccharomyces cerevisiae* for endolysin delivery to combat *Listeria monocytogenes*. Appl Microbiol Biotechnol 109(1):81. https://doi.org/10.1007/S00253-025-13464-8
